# Dietary quality indices and recurrent chronic kidney disease in Taiwanese post-renal transplant recipients

**DOI:** 10.3389/fnut.2022.1023000

**Published:** 2023-01-09

**Authors:** I-Hsin Lin, Te-Chih Wong, Tuyen Van Duong, Shih-Wei Nien, I-Hsin Tseng, Hsu-Han Wang, Yang-Jen Chiang, Shwu-Huey Yang

**Affiliations:** ^1^Department of Medical Nutrition Therapy, Linkou Chang Gung Memorial Hospital, Taoyuan, Taiwan; ^2^School of Nutrition and Health Sciences, College of Nutrition, Taipei Medical University, Taipei, Taiwan; ^3^Department of Nutrition and Health Sciences, Chinese Culture University, Taipei, Taiwan; ^4^Department of Urology, Linkou Chang Gung Memorial Hospital, Taoyuan, Taiwan; ^5^School of Medicine, Chang Gung University, Taoyuan, Taiwan; ^6^Research Center of Geriatric Nutrition, College of Nutrition, Taipei Medical University, Taipei, Taiwan; ^7^Nutrition Research Center, Taipei Medical University Hospital, Taipei, Taiwan

**Keywords:** dietary quality, kidney function, chronic kidney disease, renal transplant recipients, Taiwan

## Abstract

**Background:**

This study investigated the association between dietary quality indices and recurrent chronic kidney disease (rCKD) in Taiwanese post-renal transplant recipients (RTRs).

**Methods:**

This prospective study recruited RTRs aged >18 years with a functioning allograft and without any acute rejection in the past 3 months from September 2016 to June 2018. Dietary quality indices included the Alternative Healthy Eating Index (AHEI) and AHEI-2010, and the Taiwanese version of the AHEI (AHEI-Taiwan) was calculated using 3-day dietary records, and calculated scores were divided into quartiles. Laboratory data were collected from medical records. rCKD was defined as an estimated glomerular filtration rate (eGFR) of <60 mL/min/1.73 m^2^. Logistic regression analysis was performed to analyze the associations.

**Results:**

This study included 102 RTRs. The RTRs with higher AHEI, AHEI-Taiwan, and AHEI-2010 scores were older and had higher eGFRs and lower odds of rCKD. As compared with the lowest quartile, patients with the highest quartiles of the AHEI [odds ratio (OR), 0.10; 95% confidence interval (95% CI): 0.02, 0.49; *p*-trend = 0.004), AHEI-2010 (OR, 0.17; 95% CI: 0.04, 0.72; *p*-trend = 0.016], and AHEI-Taiwan (OR, 0.13; 95% CI: 0.03–0.59; *p*-trend = 0.008) had lower odds of rCKD, respectively. As compared with the lowest quartile, patients who consumed the highest quartiles of red and processed meat had 11.43 times higher odds of rCKD (OR, 11.43; 95% CI: 2.30–56.85; *p* for trend <0.01).

**Conclusion:**

Higher dietary quality indices are associated with lower odds of rCKD in Taiwanese RTRs. Particularly, a positive association between a higher intake of red meat and processed meat and higher odds of rCKD remained exists after transplantation in Taiwanese RTRs. Further dietary guidelines and individualized dietary education were necessary for RTRs to prevent graft function deterioration.

## 1. Introduction

Chronic kidney disease (CKD) was a major global public health problem, and its prevalence is 10–15% worldwide (1) and 11.3% in Taiwan (2). Among renal replacement therapies, renal transplantation was around 2,000 cases in Taiwan (2), which was more favorable compared with dialysis for patients with end-stage renal disease and those requiring dialysis because it had lower medical costs and results in better quality of life and higher survival rates (3). However, the elimination of dietary restrictions and conflict dietary recommendation and habits from dialysis to transplantation may also result in graft function deterioration and cause recurrent chronic kidney disease (rCKD) in renal transplant recipients (RTRs) (4).

Evidence indicates that lifestyle modifications including improved dietary quality can prevent metabolic abnormalities and reduce the risk of CKD (5, 6) and chronic diseases (7). In a previous study, we observed that RTRs had poor adherence to dietary recommendations and the intake of most nutrients was inadequate (8). The National Kidney Foundation (NKF) and National Health and Research Institutes in Taiwan (2, 9) published healthy guideline recommendations as a balance diet for RTRs includes foods from food guides, such as a variety of fresh fruits and vegetables, wholegrains, lean meats, low-fat dairy, and also low salt and high in fiber intake.

The Alternative Healthy Eating Index (AHEI) (10) includes food and nutrient components, such as trans fatty acid, the ratio of polyunsaturated fatty acid and saturated fatty acid (PSR), fruit, vegetables, wholegrains, the ratio of white and red meat, nut and soybean, and vitamin used and alcohol intake and is commonly used for the assessment of dietary quality. Both the AHEI and its updated version, AHEI-2010 (11), are based on the American Dietary Guidelines. AHEI-2010 was according to AHEI and was modified PSR to polyunsaturated fatty acid (PUFA) and n-3 PUFA, meanwhile focusing on red meat, sodium, and sugar intake. The previous study demonstrated that adherence to the AHEI and AHEI-2010 was associated with a lower risk of chronic diseases (12–14). However, Mccullough and Willett (15) reported that the dietary index can be modified according to the national dietary recommendations to be more approached to dietary culture in each country. The Taiwanese version of the AHEI-Taiwan (16) was composed as the original AHEI and modified the cutoff based on Taiwan’s dietary recommendations to adapt to Taiwanese dietary pattern.

In addition, several studies had reported that the adherence to the AHEI and AHEI-2010 was associated with decreased kidney function deterioration in CKD populations (6, 17, 18). However, the association between these indices, especially the AHEI-Taiwan, and graft function prevention had not been examined for Taiwanese RTRs. This study aimed to investigate the association between dietary quality indices and graft dysfunction in Taiwanese RTRs. We hypothesized that RTRs with higher AHEI, AHEI-Taiwan, and AHEI-2010 scores would have a lower risk of rCKD. Moreover, we explored the association between the dietary indices food component and the rCKD risk for further analysis.

## 2. Materials and methods

### 2.1. Study design and participants

This prospective cross-sectional study was conducted between September 2016 and June 2018 at Linkou Chang Gung Memorial Hospital. Inclusion criteria included that RTRs aged >18 years with a functioning allograft and without any acute rejection reaction in the past 3 months were recruited in this study. Excluded criteria included Patients with an estimated glomerular filtration rate (eGFR) variation of >25% in the past 3 months and other systemic inflammatory diseases.

A total of 106 eligible RTRs were enrolled and referred to qualified registered dietitians in the hospital for face-to-face interviews. Informed consent was obtained from each participant before the interview. The RTRs with considerably low-calorie or high-calorie intake (≤800 or ≥3,000 kcal) were excluded (*n* = 4). Hence, 102 RTRs were included in the final analysis. The study procedures complied with ethical standards for research with human participants, and the study protocol was reviewed and approved by the Institutional Review Board of Chang Gung Medical Foundation (IRB No. 201600954B0).

### 2.2. Characteristics, laboratory data, and rCKD definition

We collected the following patient characteristics and laboratory data from medical records: age, sex, dialysis history, transplant history, years after dialysis or transplantation, body height (without shoes), body weight (two times, tenth of a point taken, no shoes, and wear light clothing), performance of handgrip (measure three times for maximum values), blood pressure (average of three times), fasting plasma glucose, homeostatic model assessment of insulin resistance (HOMA-IR), total cholesterol (TC), low-density lipoprotein cholesterol (LDL-C), high-density lipoprotein cholesterol (HDL-C), triglyceride (TG), serum albumin, serum creatinine (Cr), estimated glomerular filtration rate, and high sensitive C-reactive protein. rCKD was defined as the deterioration of kidney function to end-stage renal disease (ESRD) after transplantation and was at risk for reverting to ESRD, which eGFR of <60 mL/min/1.73 m^2^ based on the Kidney Disease Outcomes Quality Initiative (KDOQI) guidelines (9).

### 3.3. Dietary intake

Dietary intake was determined using self-reported 3-day dietary records (including 2 weekdays and 1 day on the weekend) and evaluated by the qualified registered dietitian one time during the latest clinical follow-up in the study period. Dietary food and nutrient intakes were calculated using nutrition analysis software (CofitPro version 1.0.0. Cofit HealthCare Inc., Taipei, Taiwan), according to Taiwan’s Ministry of Health and Welfare Food and Drug Administration database as described previously (19).

### 3.4. Scoring of dietary quality

A total of three dietary indices based on food and nutrients were used to evaluate dietary quality: the AHEI (10), AHEI-2010 (11), and AHEI-Taiwan (16). The AHEI is based on the 2015–2020 Dietary Guidelines for Americans and includes nine components; the total AHEI score ranges from 0 (unhealthy eating quality) to 87.5 (healthy eating quality). Intermediate intake was proportionally calculated between the range of 0 and 10 points. The AHEI scores are based on the consumption of trans fat, the polyunsaturated-to-saturated fatty acid ratio (PSR) vegetables, fruits, nuts, and soybean, white and red meat, wholegrain fiber, daily multivitamins, and alcohol.

The AHEI-2010 is an updated version of the AHEI and includes 11 components; its total score ranges from 0 (unhealthy eating quality) to 110 (healthy eating quality). Compared with the AHEI, the AHEI-2010 considers the low consumption of sodium (10 points for the lowest decile) and sugar-rich beverages (10 points for <1 serving/day), the ratio of white meat to red/processed meat (10 points for 0 serving/day and 0 points for ≥1.5 servings/day), PUFA (10 points for ≥10% PUFA consumption), and n-3 PUFA (10 points for 250 mg). Moreover, in the AHEI-2010, the cutoff values were revised for the high consumption of wholegrain fiber (10 points for ≥90 g in men and ≥75 g in women) and the moderate consumption of alcohol (10 points for 0.5–3.5 equivalent in men and 0.5–2.5 equivalent in women).

The AHEI-Taiwan was developed from the AHEI according to Taiwan’s dietary recommendations for the convenience of a clinical study and better adaption to the Taiwanese population. Similar to the AHEI, the AHEI-Taiwan includes nine components, and its scores ranged from 0 (unhealthy eating quality) to 87.5 (unhealthy eating quality). In the AHEI-Taiwan, the consumption of trans fat is calculated in grams (10 points for ≥1 g and 0 points for ≤8 g); the measures for the high consumption of vegetables and fruits are revised from 5 and 4 servings/day to 3 and 2 servings/day, respectively; and the consumption of wholegrain cereal was calculated as the total recommended percent intake of cereals in Taiwan. These calculations differ from those in the AHEI.

### 3.5. Statistical analyses

The characteristics of the RTRs are summarized by the quartile of each dietary index score. Statistical analyses were performed using SAS (version 9.4, SAS Institute, Cary, NC, USA). Descriptive data are presented as the mean, standard deviation, interquartile range, and percentage as appropriate. Logistic regression analysis was performed to analyze associations between dietary quality and rCKD risk. The possible affecting factors of kidney function, such as age, sex, calorie intake, Charlson comorbidity index (CCI), body mass index, geriatric nutrition risk index, handgrip, transplantation time, and dialysis time, were adjusted based on KDOQI guidelines (9). Study data are presented as odds ratio (OR) with 95% confidence interval (95% CI). The significance was set at *P* < 0.05.

## 4. Results

### 4.1. Comparison of characteristics between those in the lowest and the highest quartile

The mean scores of AHEI, AHEI-Taiwan, and AHEI-2010 were 43.6 ± 8.8, 44.6 ± 9.0, and 62.1 ± 10.2, respectively. The RTRs in the highest quartile of both the AHEI and AHEI-Taiwan were older (53.1 ± 14.3 vs. 40.8 ± 11.5, *p* < 0.001; 51.7 ± 14.6 vs. 42.1 ± 10.7, *p* < 0.05, respectively), had higher eGFRs (64.9 ± 10.3 vs. 48.7 ± 15.9, *p* < 0.01; 64.6 ± 19.7 vs. 48.5 ± 14.8, *p* < 0.01, respectively), and had lower body height (158.7 ± 7.6 vs. 165.5 ± 8.6, *p* < 0.01; 159.1 ± 8.1 vs. 166.7 ± 8.4, *p* < 0.01, respectively), TC (196.7 ± 42.0 vs. 221.2 ± 40.8, *p* < 0.05; 195.6 ± 41.4 vs. 217.5 ± 38.2, *p* < 0.05, respectively), LDL-C (111.3 ± 38.7 vs. 135.2 ± 34.3, *p* < 0.05; 108.8 ± 38.6 vs. 134.0 ± 32.9, *p* < 0.05, respectively), and Cr (1.1 ± 0.4 vs. 1.7 ± 1.0, *p* < 0.001; 1.2 ± 0.4 vs. 1.7 ± 1.0, *p* < 0.01, respectively) levels. A greater proportion of patients in the highest quartile of the AHEI-2010 were women and older (52.8 ± 13.7 vs. 41.0 ± 10.4, *p* < 0.001) and had higher eGFRs (61.4 ± 23.6 vs. 50.9 ± 18.4, *p* < 0.05), longer transplant time (10.4 ± 5.5 vs. 7.1 ± 4.4, *p* < 0.05), and lower body height (160.0 ± 8.6 vs. 166.4 ± 9.0, *p* < 0.05) and Cr (1.3 ± 0.7 vs. 1.8 ± 1.4, *p* < 0.01) levels. The albumin level was normal in both the lowest and highest quartiles of the AHEI-2010 group (4.2 ± 0.3 vs. 4.4 ± 0.3, *p* < 0.01), but the higher albumin level was significant higher in the highest quartiles of the AHEI-2010 (Table 1).

**TABLE 1:** Comparison of the components and scores of the AHEI and AHEI-Taiwan between the lowest and highest quartiles of dietary scores (*n* = 102)^1^.

	All	AHEI		AHEI-Taiwan		AHEI-2010	
Item		Q1: 26.4-37.0	Q4: 49.3-63.2	Q1: 26.7-37.7	Q4: 51.3-68.2	Q1: 37.6-55.7	Q4: 68.3-98.8
	Mean, SD or n,%	Mean, SD or n,%	Mean, SD or n,%	Mean, SD or n,%	Mean, SD or n,%	Mean, SD or n,%	Mean, SD or n,%
Number, n	102	26	25	25	25	26	26
Age, year	48.9 ± 12.8	40.8 ± 11.5	53.1 ± 14.3^‡^	42.1 ± 10.7	51.7 ± 14.6*	41.0 ± 10.4	52.8 ± 13.7^‡^
Male, n (%)	59 (57.8)	17 (65.4)	12 (48.0)	18 (72.0)	14 (56.0)	20 (76.9)	14 (53.8)*
Cadaveric, n (%)	83 (81.3)	20 (76.9)	23 (92.0)	19 (76.0)	24 (96.0)	19 (73.1)	22 (84.6)
RT, year	8.5 ± 5.8	6.7 ± 4.2	6.2 ± 4.2	6.8 ± 4.7	5.8 ± 3.6	7.1 ± 4.4	10.4 ± 5.5*
DT, year	6.6 ± 4.9	0.8 ± 0.4	0.9 ± 0.3	0.7 ± 0.5	0.9 ± 0.3	6.6 ± 3.7	5.5 ± 3.9
WC, cm	83.1 ± 9.7	82.8 ± 10.4	81.5 ± 7.9	84.5 ± 10.9	83 ± 8.0	86.8 ± 10.6	83.1 ± 8.8
BH, cm	162 ± 8.6	165.5 ± 8.6	158.7 ± 7.6^†^	166.7 ± 8.4	159.1 ± 8.1^†^	166.4 ± 9.0	160.0 ± 8.6*
BW, kg	63.1 ± 13	64.7 ± 15.1	60.3 ± 9.2	67.2 ± 15.7	61.3 ± 9.7	69.5 ± 14.7	64.2 ± 12.2
BMI, kg/m^2^	23.9 ± 3.7	23.5 ± 4.5	23.9 ± 2.9	24.1 ± 4.7	24.1 ± 3.0	24.9 ± 4.0	24.9 ± 3.3
FPG, mg/dL	127.6 ± 24.2	121.3 ± 17.9	129.9 ± 22.9	126.5 ± 23.7	132.7 ± 24	126.8 ± 28.8	132 ± 24.2
HOMA	2.3 ± 4.5	1.7 ± 1.3	2.1 ± 1.7	3.7 ± 8.9	2.2 ± 1.7	2.1 ± 1.6	3.8 ± 8.5
TC, mg/dL	205.8 ± 43.9	221.2 ± 40.8	196.7 ± 42.0*	217.5 ± 38.2	195.6 ± 41.4*	213.5 ± 38.5	203.6 ± 45.2
LDL-C, mg/dL	119.8 ± 36.6	135.2 ± 34.3	111.3 ± 38.7*	134.0 ± 32.9	108.8 ± 38.6*	130.3 ± 33.6	116.4 ± 36
HDL-C, mg/dL	52 ± 17.9	55 ± 16.8	52.7 ± 15.2	51.2 ± 16.1	50.4 ± 16.9	53.3 ± 16.8	48.8 ± 16.4
TG, mg/dL	160.2 ± 121.6	142 ± 89.7	135.3 ± 62.8	153.7 ± 98	161.4 ± 112.1	149.5 ± 95.7	164.7 ± 86.2
Alb, g/dL	4.3 ± 0.3	4.4 ± 0.3	4.3 ± 0.3	4.4 ± 0.3	4.3 ± 0.3	4.4 ± 0.3	4.2 ± 0.3^†^
Cr, mg/dL	1.5 ± 0.9	1.7 ± 1.0	1.1 ± 0.4^‡^	1.7 ± 1.0	1.2 ± 0.4^†^	1.8 ± 1.4	1.3 ± 0.7^†^
eGFR, ml/min/1.73 m^2^	54.9 ± 20.9	48.7 ± 15.9	64.9 ± 19.3^†^	48.5 ± 14.8	64.6 ± 19.7^†^	50.9 ± 18.4	61.4 ± 23.6*
Hs-CRP, mg/dL	5.1 ± 11.4	4.1 ± 3.9	4.3 ± 5.5	3.6 ± 4.0	4.0 ± 5.5	4.9 ± 5.7	4.3 ± 5.4

^1^Value expressed as mean ± SD and percentages as appropriate. *p < 0.05, ^†^*p* < 0.01, and ^‡^*p* < 0.001 by using Student’s *t*-test or Wilcoxon rank-sum test.

Q, quartile; AHEI, Alternative Health Eating Index; SD, standard deviation; RT, renal transplant time; DT, dialysis time; WC, waist circumference; BH, body height; BW, body weight; BMI, body mass index; FPG, fasting plasma glucose; HOMA, homeostasis model assessment-insulin resistance index; TC, total cholesterol; LDL-C, low-density lipoprotein cholesterol; HDL-C, high-density lipoprotein cholesterol; TG, triglyceride; eGFR, estimated glomerular filtration rate; Hs-CRP, high-sensitivity C-reactive protein.

### 4.2. Comparison of dietary quality between the lowest and highest quartiles

The RTRs with the highest AHEI and AHEI-Taiwan scores had higher scores for the consumption of fruits (4.7 ± 2.7 vs. 1.1 ± 1.6; 7.9 ± 2.4 vs. 1.6 ± 3.0, *p* < 0.001, respectively), vegetables (6.4 ± 2.9 vs. 4.2 ± 1.7, *p* < 0.05; 8.9 ± 1.7 vs. 6.0 ± 2.1, *p* < 0.001, respectively), wholegrain (8.4 ± 3.6 vs. 0.8 ± 2.7, *p* < 0.001; 5.2 ± 5.1 vs. 0.5 ± 1.1, *p* < 0.001, respectively), white and red meat (4.8 ± 3.6 vs. 2.0 ± 1.7, *p* < 0.05; 4.9 ± 3.5 vs. 1.7 ± 1.6, *p* < 0.001, respectively), and nuts and soybean (9.1 ± 2.4 vs. 3.3 ± 4.2, *p* < 0.001; 7.8 ± 3.4 vs. 2.5 ± 3.5, *p* < 0.001, respectively) as well as higher total dietary scores (Table 2). The RTRs with the highest AHEI-2010 scores had higher scores for the consumption of fruits (4.9 ± 2.5 vs. 1.1 ± 1.6, *p* < 0.001), vegetables (6.3 ± 3.1 vs. 4.0 ± 1.7, *p* < 0.01), wholegrain (4.9 ± 4.2 vs. 0.6 ± 1.4, *p* < 0.001), and nuts and soybean (9.1 ± 2.4 vs. 4.0 ± 4.5, *p* < 0.01) and lower scores for the consumption of red or processed meat (2.4 ± 3.1 vs. 0.0 ± 0.0, *p* < 0.001), sodium (7.9 ± 2. vs. 3.7 ± 3.5, *p* < 0.001), and sugar (9.7 ± 0.4 vs. 9.4 ± 0.7, *p* < 0.05; Table 3).

**TABLE 2:** Comparison of the components and scores between the lowest and the highest quartiles of AHEI and AHEI-Taiwan dietary scores^1^.

Item	AHEI scores		AHEI-Taiwan scores	
	Q1: 26.4–37.0	Q4: 49.3–63.2	Q1: 26.7–37.7	Q4: 51.3–68.2
Trans fat,% or g^a^	10.0 ± 0.0	10.0 ± 0.0	10.0 ± 0.0	10.0 ± 0.0
PSR^b^	9.1 ± 1.7	9.9 ± 0.3	9.0 ± 1.8	9.9 ± 0.3
Fruit, servings^c^	1.1 ± 1.6	4.7 ± 2.7^‡^	1.6 ± 3.0	7.9 ± 2.4^‡^
Vegetable, servings^d^	4.2 ± 1.7	6.4 ± 2.9*	6.0 ± 2.1	8.9 ± 1.7^‡^
Wholegrain, g or %^e^	0.8 ± 2.7	8.4 ± 3.6^‡^	0.5 ± 1.1	5.2 ± 5.1^‡^
White and red meat, servings^f^	2.0 ± 1.7	4.8 ± 3.6*	1.7 ± 1.6	4.9 ± 3.5^†^
Nut and soybean, servings^g^	3.3 ± 4.2	9.1 ± 2.4^‡^	2.5 ± 3.5	7.8 ± 3.4^‡^
Vitamin used, >5 years^h^	2.5 ± 0.0	2.5 ± 0.0	2.5 ± 0.0	2.5 ± 0.0
Alcohol, equivalent^i^	0.0 ± 0.0	0.0 ± 0.0	0.0 ± 0.0	0.0 ± 0.0
Total score^j^	32.9 ± 2.8	55.8 ± 4.2^‡^	33.9 ± 2.8	57.1 ± 4.8^‡^

^1^Value expressed as mean ± SD. *p < 0.01, ^†^*p* < 0.001, and ^‡^*p* < 0.0001 by using Student’s *t*-test or Wilcoxon rank-sum test.

Q, quartile; AHEI, Alternative Health Eating Index; PSR, polyunsaturated-to-saturated fatty acid ratio; SD, standard deviation.

^a^*Trans*-fat consumption was calculated in percentage for the AHEI (10 points for ≤0.5% and 0 points for ≥4%) and in grams for the AHEI-Taiwan (10 points for ≤1 g and 0 points for ≥8 g).

^b^PSR consumption was assigned 0–10 points for a ratio <0.1 to ≥1 in both the indices.

^c^Fruit consumption was defined as follows: AHEI (0–10 points for 0–4 servings/day) and AHEI-Taiwan (0–10 points for 0–2 servings/day).

^d^Vegetable consumption was defined as follows: AHEI (0–10 points for 0–5 servings/day) and AHEI-Taiwan (0–10 points for 0–3 servings/day).

^e^Wholegrain consumption was calculated in grams for the AHEI (0–10 points for 0–15 g/day) and percentage for the AHEI-Taiwan (10 points for ≥50% of wholegrain intake).

^f^White-to-red meat ratio was assigned 0–10 points for 0–4 servings/day in both indices.

^g^Nut and soybean consumption was assigned 0–10 points for 0–1 servings/day in both the indices.

^h^Vitamin consumption was assigned 2.5–7.5 points for <5 years to ≥5 years in both the indices.

^i^Alcohol consumption was defined as 0–10 points for 0 or >3.5 equivalent and 0.5–2.5 equivalent in men and 0 or >2.5 equivalent and 0.5–1.5 equivalent in women.

^j^The total score was 2.5–87.5 in both the indices.

**TABLE 3:** Comparison of components and scores between the lowest and the highest quartiles of AHEI-2010 dietary scores^1^.

Item	Q1: 37.6–55.7	Q4: 68.3–98.8
	Score	Score
Trans fat,%^a^	10.0 ± 0.0	10.0 ± 0.0
n3-PUFA, mg^b^	8.2 ± 2.5	9.5 ± 1.5
PUFA,%^b^	9.2 ± 2.2	9.9 ± 0.2
Fruit, servings^c^	1.1 ± 1.6	4.9 ± 2.5^‡^
Vegetable, servings^d^	4.0 ± 1.7	6.3 ± 3.1*
Wholegrain, servings^e^	0.6 ± 1.4	4.9 ± 4.2^‡^
Red meat, servings^f^	0. ± 0.0	2.4 ± 3.1^‡^
Nut and soybean, servings^g^	4.0 ± 4.5	9.1 ± 2.4^†^
Alcohol, equivalent^h^	0.0 ± 0.0	0.3 ± 1.4
Na, mg^i^	3.7 ± 3.5	7.9 ± 2.1^‡^
Sugar, g^j^	9.1 ± 0.7	9.7 ± 0.4*
AHEI-2010^k^	50.0 ± 4.5	74.9 ± 6.9^‡^

^1^Value expressed as mean ± SD. *p < 0.01, ^†^*p* < 0.001, and ^‡^*p* < 0.0001 by using Student’s *t*-test or Wilcoxon rank-sum test.

Q, quartile; AHEI, Alternative Healthy Eating Index; PSR, polyunsaturated-to-saturated fatty acid ratio; SD, standard deviation; PUFA, polyunsaturated fatty acid.

^a^*Trans*-fat consumption was assigned 0–10 points for ≥4% and ≤0.5%/day.

^b^PSR consumption was assigned 0–10 points for a ratio of <0.1 to ≥1.

^c^Fruit consumption was assigned 0–10 points for 0–4 servings/day.

^d^Vegetable consumption was assigned 0–10 points for 0–5 servings/day.

^e^Wholegrain consumption was assigned 0–10 points for 0–90 g/day in men and 0–75 g/day in women.

^f^Red meat consumption was assigned 0–10 points for 1.5 and 0 servings/day.

^g^Nut and soybean consumption was assigned 0–10 points for 0–1 servings/day.

^h^Alcohol consumption was assigned 0–10 points for 0 or >3.5 equivalent and 0.5–3.5 equivalent in men and 0 or >2.5 equivalent and 0.5–2.5 equivalent in women.

^i^Sodium intake was defined as decile (0–10 points indicated the highest and lowest decile).

^j^Sugar intake was assigned 0–10 points for ≥1 (240 g) and 0 servings/day.

^k^Total score was 0–110 in both the indices.

### 4.3. Association among dietary quality, component, and rCKD

A total of 65 RTRs (64%) were diagnosed as having rCKD. All the dietary quality scores were associated with lower odds of rCKD. Compared with the lowest quartile, the highest quartile of the AHEI, AHEI-Taiwan, and AHEI-2010 had 88% (OR, 0.12; 95% CI: 0.03–0.46; *p*-trend <0.01), 87% (OR, 0.13; 95% CI: 0.03–0.48; *p*-trend <0.01), and 82% (OR, 0.18; 95% CI: 0.05–0.61; *p*-trend <0.01) lower odds of rCKD, respectively, in the crude model. In model 1, after adjustment for age and sex, the highest quartile of the AHEI, AHEI-Taiwan, and AHEI-2010 had 90% (OR, 0.09; 95% CI: 0.02–0.40; *p*-trend <0.01), 89% (OR, 0.11; 95% CI: 0.03–0.45; *p*-trend <0.01), and 85% (OR, 0.15; 95% CI: 0.04–0.59; *p*-trend <0.01) lower odds of rCKD, respectively. After additional adjustment for calorie intake and CCI, the RTRs in the highest quartile of the AHEI, AHEI-Taiwan, and AHEI-2010 had 90% (OR, 0.09; 95% CI: 0.02–0.39; *p*-trend <0.01), 90% (OR, 0.10; 95% CI: 0.03–0.43; *p*-trend <0.01), and 85% (OR, 0.15; 95% CI: 0.04–0.57; *p*-trend <0.05) lower odds of rCKD, respectively. In model 3, after further adjustment for body mass index (BMI), geriatric nutrition risk index (GNRI), handgrip, transplant time, and dialysis time, the RTRs in the highest quartile of the AHEI, AHEI-Taiwan, and AHEI-2010 had 91% (OR, 0.09; 95% CI: 0.02–0.46; *p*-trend <0.01), 88% (OR, 0.12; 95% CI: 0.03–0.59; *p*-trend <0.01), and 83% (OR, 0.17; 95% CI: 0.04–0.73; *p*-trend = 0.02) lower odds of rCKD, respectively (Table 4). Further analysis revealed that RTRs who consumed high amounts of red/processed meat had 11.43 times higher odds of rCKD (OR, 11.43; 95% CI: 2.30–56.85; *p*-trend <0.01; Table 5).

**TABLE 4:** Risk of incident chronic kidney disease by the AHEI, AHEI-Taiwan, and AHEI-2010 in the renal transplant recipients^1^.

Item	Q1	Q2	Q3	Q4	*P* trend
**AHEI**					
Crude	1 (reference)	0.23 (0.06–0.87)	0.49 (0.13–1.95)	0.12 (0.03–0.46)	0.002
Model 1	1 (reference)	0.19 (0.05–0.77)	0.38 (0.09–1.62)	0.09 (0.02–0.40)	0.001
Model 2	1 (reference)	0.19 (0.05–0.79)	0.34 (0.08–1.48)	0.09 (0.02–0.39)	0.001
Model 3	1 (reference)	0.15 (0.03–0.79)	0.38 (0.08–1.87)	0.09 (0.02–0.46)	0.003
**AHEI-Taiwan**					
Crude	1 (reference)	0.52 (0.13–2.05)	0.26 (0.07–0.98)	0.13 (0.03–0.48)	0.003
Model 1	1 (reference)	0.50 (0.12–2.04)	0.22 (0.05–0.89)	0.11 (0.03–0.45)	0.002
Model 2	1 (reference)	0.44 (0.10–1.80)	0.20 (0.05 - 0.82)	0.10 (0.03–0.43)	0.002
Model 3	1 (reference)	0.43 (0.09–2.11)	0.26 (0.06–1.23)	0.12 (0.03–0.59)	0.009
**AHEI-2010**					
Crude	1 (reference)	0.51 (0.14–1.83)	0.42 (0.12–1.51)	0.18 (0.05–0.61)	0.006
Model 1	1 (reference)	0.50 (0.13–2.00)	0.37 (0.10–1.44)	0.15 (0.04–0.59)	0.006
Model 2	1 (reference)	0.54 (0.13–2.16)	0.32 (0.08–1.29)	0.15 (0.04–0.57)	0.006
Model 3	1 (reference)	0.42 (0.09–2.09)	0.33 (0.07–1.52)	0.17 (0.04–0.73)	0.02

^1^Value expressed as OR (95% CI) by using logistic regression, Model 1 was adjusted for age and sex. Model 2 was adjusted for age, sex, calorie intake, and CCI. Model 3 was adjusted for age, sex, calorie intake, CCI, BMI, GNRI, handgrip, transplant time, and dialysis time.

Q, quartile; AHEI, Alternative Healthy Eating Index; CCI, Charlson comorbidity index; BMI, body mass index; GNRI, geriatric nutrition risk index.

**TABLE 5:** Risk of incident chronic kidney disease by dietary indices in the renal transplant recipients.

Item	Q1	Q2	Q3	Q4	*P* trend
**Fruits, servings**					
Crude	1 (Reference)	0.77 (0.22–2.73)	0.21 (0.06–0.68)	0.60 (0.17–2.09)	0.42
Model 1	1 (Reference)	0.89 (0.24–3.28)	0.21 (0.06–0.7)	0.63 (0.17–2.29)	0.48
Model 2	1 (Reference)	0.85 (0.23–3.22)	0.18 (0.05–0.64)	0.64 (0.17–2.39)	0.50
Model 3	1 (Reference)	0.68 (0.15–3.14)	0.16 (0.04–0.72)	0.80 (0.17–3.75)	0.77
**Vegetable, servings**					
Crude	1 (Reference)	0.88 (0.29–2.70)	4.12 (0.98–17.38)	0.50 (0.17–1.51)	0.22
Model 1	1 (Reference)	0.86 (0.28–2.65)	0.93 (0.29–2.97)	1.01 (0.27–3.74)	0.23
Model 2	1 (Reference)	3.97 (0.93–16.86)	3.93 (0.91–17.01)	5.19 (1.08–24.96)	0.21
Model 3	1 (Reference)	0.50 (0.16–1.55)	0.49 (0.16–1.53)	0.57 (0.16–2.11)	0.40
**White and red meat ratio**					
Crude	1 (Reference)	1.18 (0.33–4.18)	0.58 (0.17–2.05)	0.33 (0.01–1.08)	0.07
Model 1	1 (Reference)	1.22 (0.34–4.36)	1.19 (0.33–4.32)	2.38 (0.55–10.32)	0.08
Model 2	1 (Reference)	0.59 (0.17–2.08)	0.58 (0.16 - 2.11)	1.46 (0.33–6.59)	0.07
Model 3	1 (Reference)	0.33 (0.10–1.12)	0.31 (0.09–1.08)	0.50 (0.13–1.96)	0.32
**Red/processed meat, servings**					
Crude	1 (Reference)	2.98 (0.93–9.57)	3.89 (1.21–12.55)	8.75 (2.20–34.81)	0.002
Model 1	1 (Reference)	3.08 (0.95–10.05)	3.86 (1.13–13.23)	3.59 (0.94–13.69)	0.003
Model 2	1 (Reference)	4.10 (1.19–14.1)	4.87 (1.34–17.71)	4.06 (0.97–16.97)	0.001
Model 3	1 (Reference)	8.83 (2.13–36.61)	11.54 (2.68–49.77)	11.43 (2.30–56.85)	0.003
**Nut and soybeans, servings**					
Crude	1 (Reference)	2.00 (0.47–8.59)	0.57 (0.21–1.58)	0.83 (0.28–2.50)	0.75
Model 1	1 (Reference)	2.30 (0.51–10.34)	2.10 (0.46–9.58)	2.86 (0.56–14.74)	0.84
Model 2	1 (Reference)	0.53 (0.19–1.52)	0.52 (0.18–1.49)	0.47 (0.14–1.56)	0.77
Model 3	1 (Reference)	0.89 (0.28–2.79)	0.84 (0.26–2.69)	0.79 (0.21–2.90)	0.72

^1^Value expressed as OR (95% CI) by using logistic regression. Model 1 was adjusted for age and sex. Model 2 was adjusted for age, sex, calorie intake, and CCI. Model 3 was adjusted for age, sex, calorie intake, CCI, BMI, GNRI, handgrip, transplant time, and dialysis time.

Q, quartile; CCI, Charlson comorbidity index; BMI, body mass index; GNRI, geriatric nutrition risk index.

## 5. Discussion

The results of this cohort study revealed that the RTRs in the highest quartiles of the AHEI, AHEI-Taiwan, and AHEI-2010 had 91, 88, and 83% lower odds of rCKD, respectively, compared with the RTRs in the lowest quartiles of these indices after adjustment for age, sex, calorie intake, CCI, BMI, GNRI, handgrip, transplant time, and dialysis time. In addition, higher consumption of red/processed meat was associated with 11.4 times higher odds of rCKD.

The results of the present study are consistent with those of a prospective cohort study (20) with a follow-up period of 14.3 years that recruited 4,848 participants and examined their dietary quality by using the Health Eating Index (HEI), AHEI-2010, Dietary Approaches to Stop Hypertension (DASH) diet, and alternate Mediterranean diet (aMED) and indicated that high dietary quality was associated with CKD prevention. Hu et al. (6) included 3,980 patients with CKD with a follow-up period of 24 years and indicated that high HEI, AHEI-2010, and aMED scores were associated with a 13–20% lower risk of incident CKD. Osté et al. (21) reported that the high scores of the DASH diet were associated with lower renal dysfunction and mortality in RTRs. In addition, some studies have demonstrated that the DASH diet and aMED were inversely associated with the risk of CKD and prevented a decrease in the eGFR and an increase in Cr and micro-albuminuria levels (22–25). These findings suggest that high dietary quality is associated with CKD prevention. Notably, the prevention of rCKD is more important for RTRs due to the elimination of dietary restrictions and incorrect dietary habits after transplantation (4). On the contrary, Song et al. (26) demonstrated that a revised version, the DASH-Japan Ube Modified diet Program (DASH-JUMP) and Korean DASH diet (K-DASH) were modified according to Japanese and Korean dietary recommendation, which is consistent with the present study of AHEI-Taiwan to adapt Taiwanese dietary recommendations.

Regarding the component of dietary indices, vegetable and fruit consumption were not associated with preserving the eGFR in the present study; this finding is in agreement with that of a previous study that enrolled Dutch (27) and American (28) participants. However, Jhee et al. (29) demonstrated that the high consumption of vegetables and fruits was associated with decreased albuminuria and kidney injury. A reason for this finding is that the consumption of vegetables and fruits rich in potassium is associated with lower blood pressure, which possibly prevents kidney function deterioration (30). Another reason for the positive association between vegetable and fruit consumption and lower risk of CKD may be the effect of decreased acid load. The high dietary acid load may increase metabolic acidosis and lead to kidney injury through an increase in the levels of endothelin-1, which stimulates aldosterone production by activating the renin–angiotensin–aldosterone system pathway, increasing the ammonium concentration, and leading to kidney tubular injury, endothelial dysfunction, and inflammation (31–33). Future studies should investigate the effect of vegetable and fruit consumption on rCKD in RTRs.

Previous studies (34) have examined the association between different protein sources such as red/processed meat, nuts, and soybean, and CKD prevalence. Red/processed meat can lead to inflammation, increase sodium load and iron’s pro-oxidative effects, and cause DNA damage, thus directly or indirectly affecting kidney function. In addition, animal protein sources increase the acid load, whereas plant protein sources increase alkalosis load; the association between acid load and CKD progression has also been demonstrated (35). No associations between white-to-red meat ratio, nut and soybean consumption, and rCKD were noted. However, O’Keefe et al. (36) demonstrated that the high consumption of soybean was associated with decreased phosphate intake and urinary protein excretion, thus preventing CKD progression. Haring et al. (37) and Mirmiran et al. (38) have reported that replacing one serving of total red/processed meat with one serving of legumes, nuts, wholegrain cereal, low-fat dairy, and fish and seafood was associated with 18–31% and 16–21% lower risk of CKD, respectively. Future studies should evaluate the association between replacing protein sources and rCKD risk in RTRs.

This study has some strengths and limitations. To date, this is the first study to investigate the association between dietary quality and rCKD in Taiwanese RTRs. However, the causality could not be interpreted because of the cross-sectional design of this study. Although the findings of the current study limit the evidence of randomized controlled trials, the results were obtained using 3-day dietary records including 2 weekdays and 1 day on the weekend, and 24-h recall was used to determine dietary quality based on food composition data provided by Taiwan’s Ministry of Health and Welfare. Furthermore, a composite definition was used to define rCKD. These methods helped us assess the dietary intake of the RTRs, evaluate nutrition-related problems, and enhance awareness regarding dietary quality and the rCKD in Taiwanese RTRs. The small sample size of this study may reduce the statistical power (β = 0.7) to detect significant associations. Finally, although many potential confounders were adjusted, the possibility of imperfectly measured or unknown confounders (such as non-immunological and immunological factors) was not excluded in this observational study.

## 6. Conclusion

This prospective study examined food and nutrient intake, which reflect dietary quality in patients receiving renal transplantation. Overall, higher AHEI, AHEI-Taiwan, and AHEI-2010 scores were associated with lower odds of rCKD in Taiwanese RTRs. Notably, AHEI-Taiwan is based on Taiwan’s dietary recommendation, which may be more adaptive to Taiwanese populations. Moreover, further analysis for the dietary component as red/processed meat was positively associated with rCKD. Additional longitudinal and randomized controlled studies are required to verify the association between dietary quality and rCKD.

## Data availability statement

The original contributions presented in this study are included in this article/supplementary material, further inquiries can be directed to the corresponding author.

## Ethics statement

The studies involving human participants were reviewed and approved by the Institutional Review Board of Chang Gung Medical Foundation (IRB No. 201600954B0). The patients/participants provided their written informed consent to participate in this study.

## Author contributions

I-HL, TD, T-CW, and S-HY: conceptualization. I-HL and TD: methodology and analysis and interpretation of data. I-HL, S-WN, and I-HT: software. TD, T-CW, and S-HY: validation and supervision. I-HL, S-WN, H-HW, and Y-JC: investigation. H-HW and Y-JC: resources. I-HL, TD, S-WN, and I-HT: data curation. I-HL: visualization and writing—original draft. I-HL, TD, and S-HY: writing—reviewing and editing. I-HL, S-WN, I-HT, H-HW, and Y-JC: project administration. All authors contributed to the article and approved the submitted version.
